# Supplementary material for: Chondroitin Sulfate in USA Dietary Supplements in Comparison to Pharma Grade Products: Analytical Fingerprint and Potential Anti-Inflammatory Effect on Human Osteoartritic Chondrocytes and Synoviocytes
Source: Pharmaceutics. 2021 May 17;13(5):737. doi: 10.3390/pharmaceutics13050737 (PMC8156081; doi:10.3390/pharmaceutics13050737)
Supplement: Supplementary file 1 [file pharmaceutics-13-00737-s001.zip › pharmaceutics-1193316-supplementary.pdf]

pharmaceutics

MDPI

Supplementary Materials: Chondroitin Sulfate in USA Dietary Supplements in Comparison to Pharma Grade Products: Analytical Fingerprint and Potential Anti-Inflammatory Effect on Human Osteoartritic Chondrocytes and Synoviocytes

Antonietta Stellavato, Odile Francesca Restaino, Valentina Vassallo, Elisabetta Cassese, Rosario Finamore, Carlo Ruosi and Chiara Schiraldi

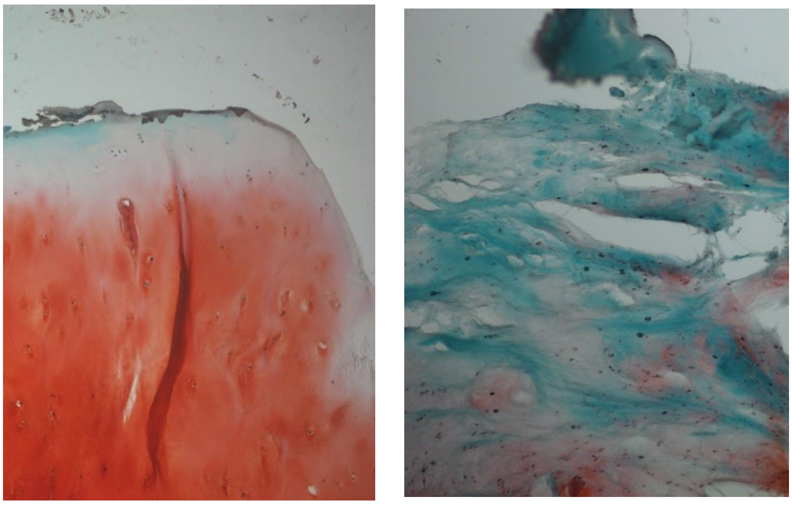

(**A**) (**B**)

Figure S1. Microscopic evaluation of Safranin-O/Fast Green Staining. Histological sections were observed under light microscopy at low magnification: 10×. Panel A is a representative of less damaged tissue while B represents a more compromised sampling area.

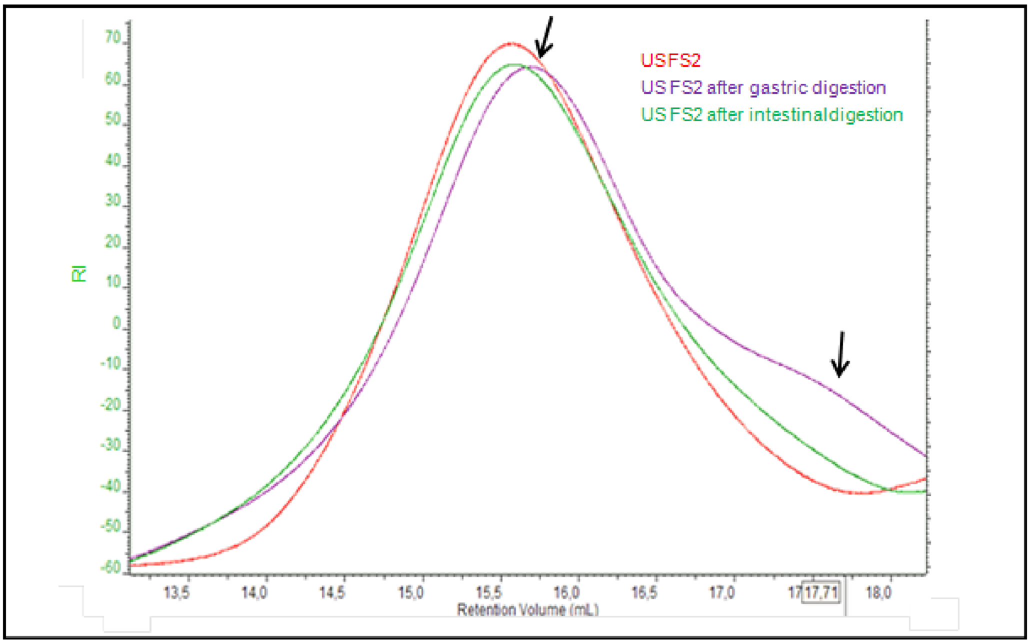

Figure S2. Representative overlaid SEC-TDA chromatograms of US food supplement before and after gastric and intestinal digestion. The two different CS molecular weight species are indicated by the arrows.

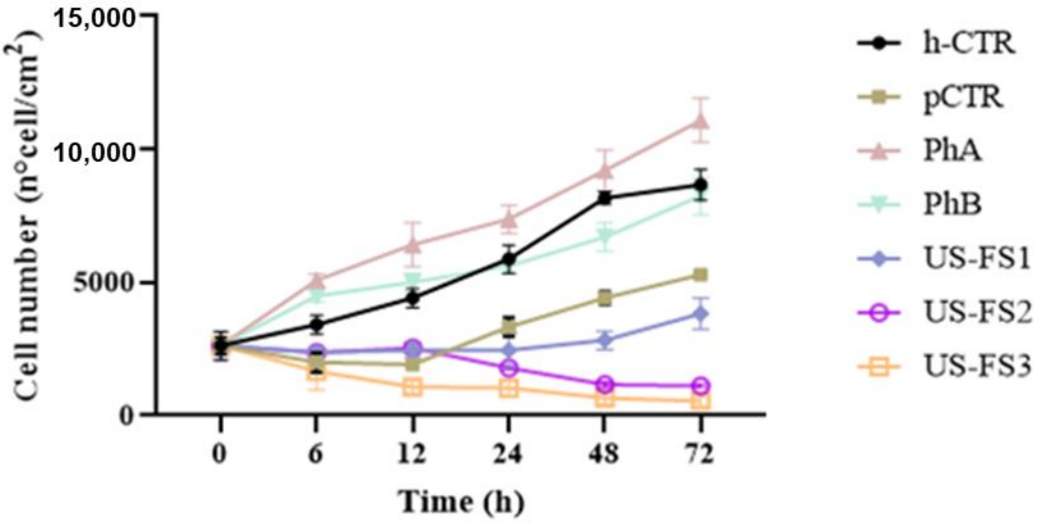

Figure S3. Cell growth and proliferation of chondrocytes treated with US-FS in comparison to PhA and PhB pharma grade chondroitin sulfate based products, using Time Lapse Video Microscopy. Figure shows growth curves obtained by data analysis of time lapse experiment performed on four field of view for each sample and for two well (in duplicate).
